# Views of patients with advanced disease and their relatives on participation in palliative care research

**DOI:** 10.1186/s12904-021-00779-2

**Published:** 2021-06-05

**Authors:** Karolina Vlckova, Kristyna Polakova, Anna Tuckova, Adam Houska, Martin Loucka

**Affiliations:** 1 Center, for Palliative Care, Dykova 15, Prague, 110 00 Czech Republic; 2 First Faculty of Medicine, Charles University, Prague, Czech Republic; 3 Third Faculty of Medicine, Charles University, Prague, Czech Republic; 4 Faculty of Social Science, Charles University, Prague, Czech Republic

**Keywords:** Research ethics, Family, Patients, Palliative care, Research subjects, Research participation

## Abstract

**Background:**

Patients with advanced disease may not be invited to participate in research based on the assumption that participation would be too burdensome for them. The aim of this study was to explore how patients with advanced disease and their relatives evaluate their experience with research participation.

**Method:**

This study used data from two parts of a larger project. The first dataset was a cross-sectional questionnaire study focused on priorities at the end of life. The second dataset used a longitudinal design with structured interviews on prognostic awareness. In both studies, participants evaluated their experience on a 5-point Likert scale and specified their motivation in an open-ended question.

Data were collected in 6 hospitals in the Czech Republic with patients with advanced disease and life expectancy less than 1 year and their relatives. Data were analysed using non-parametric tests and thematic analysis.

**Results:**

First dataset consisted of 167 patients and 102 relatives, and second dataset consisted of 135 patients and 92 relatives (in total, 496 respondents). Results were similar in both datasets, with half of the sample (53%, 48%) scoring neutral, and over 30% of the sample identified their experience as interesting. The most significant factors associated with the evaluation were religiosity (*p* = 0.001) and the type of diagnosis (*p* = 0.04). Motivation for participation was to improve care, support research, express own opinion, opportunity to talk and trusting relationship.

**Conclusions:**

Patients with advanced disease and relatives do not mind participating in palliative care research, and it can be even a positive experience for them.

**Supplementary Information:**

The online version contains supplementary material available at 10.1186/s12904-021-00779-2.

## Background

Patients in need of palliative care are often seen as too vulnerable to participate in end-of-life research, but this should not lead to the assumption that they should not be included in palliative care research [1]. With respect to their autonomy, patients should be given a choice to decide about their research participation by themselves [2, 3]. Denying patients and their family carers of this choice is deemed as unethical [1] and paternalistic [4] and can jeopardise the further development of evidence-based palliative care. Current evidence suggests that patients and their relatives have a positive attitude toward end-of-life research and describe their experience of participation in research as positive or even therapeutic [2–4]. By participating in research, patients have the opportunity to express their altruism, which was identified as one of the main reasons for their engagement with research in several studies [2, 3, 5–7]. Nevertheless, their willingness is strongly connected to the invasiveness of the study [8], which indicates a possible difference in patient's attitudes based on the study design.

The likelihood of patients and their relatives being invited to participate in end-of-life research is greatly influenced by health care professionals who act as gatekeepers [9]. In a recent systematic review, the “fear of burdening the patient” was identified as the main reason for not approaching patients in end-of-life research [9]. The urgency to protect potentially vulnerable participant means healthcare professionals may be reluctant to recruit eligible patients into a palliative care study [1, 9, 10].

To challenge this perception of patients’ participation in end-of-life research, studies focused on exploring how patients and their relatives themselves experienced their participation in research are necessary. Available studies focused on this topic originate dominantly from the USA, United Kingdom or Australia and are predominantly set in a cancer patient population [4].

The aim of this study was to explore how patients with advanced disease (both cancer and other) and their relatives feel about their participation in palliative care research. The study was a part of a three-year-long research project focused on prognostic awareness in patients with advanced cancer (Integrative model of prognostic awareness in patients with advanced cancer—IMPAC), and the results are based on two datasets from different parts of this project.

## Dataset 1

### Methods

This dataset was collected during a multicentre cross-sectional study aimed to identify the priorities of patients with advanced disease and their informal caregivers. Participants were recruited from May till September 2018 at various departments in 2 regional and 3 university hospitals in the Czech Republic. Inclusion criteria for patients were age 18 + , cognitive ability to participate, and patients’ life expectancy less than 1 year estimated by their physicians using the surprise question [11]. Eligible patients were invited to participate during hospital admission by their physicians, who informed them about the purpose of the study. Recruitment of relatives happened during their hospital visit, and they were eligible to participate if they were related to a person fulfilling the patient inclusion criteria. All participants provided written consent, and the study was approved by research ethics committee at each data collection site.

Data were collected by a questionnaire which was designed specifically for this study and was based on findings from a non-published qualitative pre-study conducted during the IMPAC project, focused on exploring the priorities of patients with advanced cancer. The results of this pre-study informed the development of the final questionnaire consisting of 40 different factors. Participants of this questionnaire study were asked to rank the factors by their importance on 5-point Likert scale. Demographic factors and the relationship of relatives to the patients were also collected. Additionally, in open-ended question, participants were asked to state their main motivation for agreeing to participate in the study and to evaluate how they felt about their experience on 5-point Likert scale (Very interesting, Interesting, I did not mind, Unpleasant, Very unpleasant). Patients had the questionnaire administrated by trained medical staff while relatives completed the questionnaire by themselves.

### Analysis

Distribution of data was analysed using Kolmogorov–Smirnov test, which found that the distribution was not normal, therefore for further analysis, non-parametric methods were used (Mann–Whitney test, Spearman's correlation). Statistical analysis was conducted in IBM SPSS 26.

Written answers to the open-ended question were analysed by two researchers independently (KP, KV) using thematic analysis approach [12]. Verbatim responses were extracted and analysed separately for patients and relatives.

### Results

The sample consisted of 170 patients and 108 relatives, but 9 respondents (3 patients, 6 relatives) were excluded from the analysis because they did not complete the question evaluating their research experience. Demographics of the final sample (*N* = 269) are reported in Table 1.

**Table 1:** Demographics of participants dataset 1

	**Patients (** ***n*** ** = 167)**	**Relatives (** ***n*** ** = 102)**
**Sex**	49% female	72% female
Mean age (SD)	69.6 (12.7)	57.8 (14.5)
**Education**		
Elementary school	14.5%	8.8%
High school	66.2%	57.8%
Graduate degree	19.3%	33.3%
**Being religious**	40%	45.5%
**Relationship to patient**		
Spouse/ Husband	NA	25.5%
Daughter / Son	NA	48%
Granddaughter / grandson	NA	9.8%
Sibling	NA	3%
Other	NA	13.7%

Half of the sample (53%) did not mind participating in this study and for almost 40% it was an interesting or very interesting experience. Detailed information is provided in Table 2.

**Table 2:** Evaluation of participation in dataset 1

	**Patients**	**Relatives**
Very unpleasant	1%	0%
Unpleasant	6%	2%
I do not mind	53%	57%
Interesting	33%	33%
Very interesting	7%	8%
N	167	103

The difference in answers of patients and relatives was not significant (*p* = 0.52). In the group of patients, the answers did not correlate with age (*R* = 0.1; *p* = 0.23) and did not differ based on gender (*p* = 0.17), being religious (*p* = 0.5), education (*p* = 0.19) or the level of prognostic awareness (*p* = 0.5). Similarly, in the group of relatives, the answers did not correlate with age (*R* = 0.1; *p* = 0.3) and did not differ based on gender (*p* = 0.25), education (*p* = 0.5) or being religious (*p* = 0.14). The answers differed based on the type of diagnosis (*p* = 0.04) because patients with noncancer diagnosis evaluated their participation positively (Median = 4) versus cancer patients (Median = 3).

### Open-ended question

The open-ended question about participants' motivation was answered by 78 patients and 42 relatives.

In the group of patients following five themes were identified: Improving care, Supporting research, Expressing own opinion, Trust, Opportunity to talk. In the relative’s group, there were four analytical themes, which were identical to the themes in the patient's group (Improving care, Supporting research, Express own opinion, Trusting relationship). These findings indicate that patient and relatives are motivated by similar aspects. Therefore, the results of the analysis are presented together.

#### Theme 1: Improving care

Improving health care for others was a major motivation for participation in both groups of respondents. Patients and relatives had a desire to help to improve not only medical care but also the relationship and communication between patients and physicians and to help others in a similar situation:

*(I have) a great interest to improve care for other patients. (Patient)*

#### Theme 2: Supporting research

Patients and relatives expressed a strong wish to support research focused on a topic they sought as important and interesting. The respondents believed that research is necessary for developing knowledge in this field, and their participation in research is thus meaningful and important.

*I like to help, and I think that the research is meaningful. (Patient)*

#### Theme 3: Expressing own opinion

The participation in research was also motivated by the wish to express their opinion. Being able to express own feelings and experiences was acknowledged as an important aspect of medical care, and respondents felt that it is important for doctors to know what they think.

*It is important to know the opinions of the closest people of the patients. (Relative)*

#### Theme 4: Trusting relationship

Patients and relatives were motivated to participate in research because they were approached by a health care staff whom they trusted and have already developed a relationship with. It was also an opportunity to express their gratitude for the care they received.

*I was approached by the doctor who is taking excellent care of my mother. (Relative)*

*Because I trust you*. (Patient).

#### Theme 5: Opportunity to talk

This theme was identified only in the patients' group. Participation in research gave patients an opportunity to talk with somebody and think about topics they otherwise would not. The desire to speak with someone was driven by the sense of loneliness and by the stereotype of their days while staying at the hospital. Answering the questionnaire helped them to explore their feelings and opinions and gave them an opportunity to get new experience.

*I am alone in the hospital room; therefore, I am glad I can speak with somebody. Maybe I will learn something new. (Patient)*

## Dataset 2

### Methods

The second dataset was collected during a multicentre longitudinal cohort study which was another part of the IMPAC project from September 2018 till September 2019 at oncology departments in three university hospitals in Prague.

The study included patients with advanced cancer and their relatives. Inclusion criteria for patients were a diagnosis of advanced cancer with limited prognosis (assessed by treating physician using the 12-month surprise question) and cognitive ability to participate in a structured interview. Relatives were invited to participate if identified by patients as their primary caregivers. Written or verbal consent was obtained from all participants. A research ethics committee approved the study at each data collection site.

Data were collected by experienced researchers who followed a structured interview protocol focused on participant's prognostic awareness and their quality of life (Integrated Palliative Outcome Scale [13]). The protocol included a question evaluating participants’ research experience (for complete questionnaire, see Additional file 1). The data collection was repeated twice over 9 months after the baseline contact. The baseline data collection with patients was conducted face-to-face at the hospital. The second and third measurements and data collection with the relatives were conducted mainly by phone.

### Analysis

Distribution of data was analysed using Kolmogorov–Smirnov test, which found that the distribution was not normal, therefore for further analysis, non-parametric methods were used (Spearman's correlation test, Mann–Whitney test, Kruskal–Wallis test, and Friedman test). Statistical analysis was conducted in IBM SPSS 26.

### Results

The study sample included 137 patients and 94 relatives. For further analysis, 4 participants (2 patients and 2 relatives) were excluded because they did not complete the question evaluating their participation in the study. Detailed demographics of the final samples are reported in Table 3.

**Table 3:** Demographics of participants dataset 2

	**Patients (** ***n*** ** = 135)**	**Relatives (** ***n*** ** = 92)**
**Sex**	44,5% female	74.5% female
Mean age (SD)	64.6 (9.2)	52.9 (12.5)
**Education**		
Elementary school	10%	5%
High school	70%	71.5%
Graduate degree	20%	24.5%
**Being religious**	50%	38%
**Relationship to patient**		
Spouse/ Husband	NA	33.6%
Daughter / Son	NA	29.2%
Granddaughter / grandson	NA	0.7%
Sibling	NA	2.9%
Other	NA	2.2%
Not available	NA	31.4%

Half of the sample of patients (48%) did not mind participating in this research, 34% found it as interesting and 17% as very interesting experience (see Table 4). The answers were not associated with sex (*p* = 0.75), education (*p* = 0.56), level of prognostic awareness (*p* = 0.89), quality of life as measured with IPOS (*R* = -0.1; *p* = 0.2) or pain (*R* = 0.05; *p* = 0.6) but were positively associated with being religious (Z = -3.4; *p* = 0.001) and slightly with older age (*R* = 0.2; *p* = 0.03).

**Table 4:** Evaluation of participation dataset 2

	**W1** **patients**	**W1** **relatives**	**W2** **patients**	**W2** **relatives**	**W3** **patients**	**W3** **relatives**
Very unpleasant	**0%**	**0%**	**0%**	**0%**	**0%**	**0%**
Unpleasant	**1%**	**3%**	**1%**	**5%**	**4.5%**	**6%**
I do not mind	**48%**	**54%**	**64%**	**57%**	**54.5%**	**71%**
Interesting	**34%**	**32%**	**22%**	**19%**	**27%**	**12%**
Very interesting	**17%**	**11%**	**13%**	**19%**	**14%**	**12%**
N	**135**	**92**	**69**	**21**	**43**	**17**

The data collection was repeated every 2–3 months, with all three measurements being completed by 33,8% of the patients’ sample. In total, 92 patients dropped out of the study for various reasons. The main reason was death (41 patients), patient’s will to quit the study (31 patients), hospice referral (7 patients), non-functional contact (17 patients) and deterioration of health (8 patients). The reason for withdrawal from the study was not possible to identify in 10 respondents. The evaluation of participation did not differ in patients who withdraw from the study (*N* = 92) from patients who completed all three measurements (*N* = 43 Z = -0.29; *p* = 0.8). In the group of patients who completed all three waves was no significant difference in the evaluation of their experience when measured over time (*N* = 43; χ2 = 2.9; *p* = 0.2).

In the sample of relatives, 53% of the respondents did not mind participating in this research, 32% found it as interesting and 11% as a very interesting experience. The answers were not associated with age (*R* = 0.01; *p* = 0.9), sex (*p* = 0.7), education (*p* = 0.85), or being religious (*p* = 0.8). Relatives evaluated their participation in the study similarly to patients, and the difference was not significant (Z = -1,4; *p* = 0.16). The dropout of relatives was bigger than in the patient group (81% in relative vs 69% in patient’s group).

## Discussion

The results of the presented study indicate that patients and relatives do not mind participating in palliative care research. Moreover, many of them describe their participation as an interesting experience. Positive attitudes towards participation in research identified in our study are consistent with previous research [8, 14–17]. This study adds new evidence that patients and relatives evaluate their participation positively even when measured over time in a longitudinal study.

The positive evaluation of participation in research might be influenced by several factors such as gender, level of education, pain, prognostic awareness, or quality of life [5, 8, 15]. In the presented study, three factors were positively associated with the experience of patients but not the relatives.

The first identified factor was the type of diagnosis. Patients with non-cancer diagnosis evaluate their experience positively versus patients with cancer. This is a very important finding because in previous studies, attitudes of cancer patients were mainly studied [7, 15]. On the other hand, this result is consistent with a previous study that showed that patients with cancer more often declined participation in research against patients with neuron disease [6]. This might be explained by the various explanations that cancer patients with a more predictable prognosis might be more distressed, or they might have other priorities. This needs to be further studied.

The second significant factor was religiosity. Participants which identified themselves as being religious evaluated their participation as more positive. On the other hand, the percentage of religious respondents was higher in our sample than is in the general Czech population. This may indicate that considering yourself as a religious person might be a moderate factor for the evaluation of research participation. Thus, this finding supports previous research identifying religious people as less stressed while participating in palliative care research [15, 18].

The third identified factor with a positive correlation with a positive evaluation of participation was higher age. This finding must be interpreted with caution as the correlation was weak, and it was identified only in the dataset from the longitudinal study. Association between research participation and age has been reported elsewhere with mixed results [5, 8, 15], with younger patients being more willing to participate in research than older patients [5, 8]. The role of age thus remains unclear and more research focused on this factor is needed.

The relatives’ evaluation of research participation was also predominantly positive which supports findings from Aoun et al. study focused on relative’s perception on participating in research with majority of them identifying their experience as beneficial [19].

Patients and relatives were motivated to participate in the presented study by several reasons, including a desire to improve medical care for others and to support research. Similar reasons which motivated patients to participate in research were identified in previous studies, with altruism being the main motive for participation [5, 6, 15]. Those findings suggest that patients have a desire to help others, and participation in research serves as an opportunity how to do this. Research participation was also perceived by patients as an opportunity for social interaction during a hospital admission which helped them to pass their time in the hospital. This is consistent with previous research in this field [2, 4, 6, 15] and suggests that timing of data collection might be crucial for successful recruitment of patients in the study, such as interviewing patient while waiting for chemotherapy. Also, being approached by a familiar person with whom the patients have already established a relationship can enhance the patient’s motivation to get involved in research [2, 14, 17].

The dropout analysis in the longitudinal study indicates the ability of participants to decide about their research participation. The dropout rate was not driven by a negative experience, on the contrary, those respondents identified research participating as an interesting endeavour. This finding supports the idea that patients with advanced disease are able to choose if they want to participate in research or not [15], and rather than protecting them on the assumption of research participation being harmful to them, they should be given a choice to make this decision for themselves.

The main strength of this study is including participants who have real experience with participation in palliative care research, also involving patients with another advanced disease than cancer and using a longitudinal design. This study also has several limitations. Patients could underreport their discomfort due to social desirability factors and because the evaluation question was administered by the same person as the whole questionnaire. The qualitative question was not answered by all participants; thus the motivation of those who did not answer could be different. The thematic analysis was done on written responses, which could lead to misinterpretation of its meaning. Additionally, the results may differ in research focused on other aspects than prognostic awareness, quality of life or patients’ preferences.

## Conclusion

This study highlights some important aspect in research with patients with advanced illness and their relatives. Most of the study participants identified their participation as an interesting experience giving them an opportunity to express their opinion and to do some good such as support research or improve care. Participation in a longitudinal study with repetitive measurements was not experienced as unpleasant, and respondents were able to withdraw from the study if it became too burdensome. The need to respect patient's autonomy should be acknowledged in research. This paper supports evidence that patients with advanced illness should be given the option to participate in research as they are able to decide for themselves.

## Supplementary Information

**Additional file 1**

## Data Availability

The datasets used during the study are available from the corresponding author on reasonable request.

## Abbreviations

IPOS: Integrated Palliative Outcome Scale
IMPAC: Integrated model of prognostic awareness in patients with advanced cancer study
