# Supplementary material for: Views of patients with advanced disease and their relatives on participation in palliative care research
Source: BMC Palliat Care. 2021 Jun 5;20:80. doi: 10.1186/s12904-021-00779-2 (PMC8180046; doi:10.1186/s12904-021-00779-2)
Supplement: Supplementary file 1 — Additional file 1 [file 12904_2021_779_MOESM1_ESM.docx]

Additional file 1

**Patient´s code ……………………………**

**Patients**

date (dd/mm/yyyy)

|  |  |  |  |  |  |  |  |
| --- | --- | --- | --- | --- | --- | --- | --- |

1. **How important is for you to have information about prognosis of your disease?**
2. Very unimportant
3. Fairly unimportant
4. Fairly important
5. Very important
6. **How would you describe your current health status?**
7. I am relatively healthy
8. I am ill, but not seriously
9. I am seriously ill but my life is not currently in danger
10. I am seriously and terminally ill
11. **How do you think that your family would describe your current health status?**
12. I am relatively healthy
13. I am ill, but not seriously
14. I am seriously ill but my life is not currently in danger
15. I am seriously and terminally ill and I will probably die soon

1. **How likely is that your disease will be cured?**

Very unlikely It is sure that I will be cured

|  |  |  |  |  |  |  |  |  |  |
| --- | --- | --- | --- | --- | --- | --- | --- | --- | --- |

0 % 50 % 100 %

1. **What do you think is currently the main goal of your oncological treatment?**
2. To cure my disease
3. To prolong my life (although the disease can no longer be cured)
4. To relieve symptoms
5. **Have you talked about the seriousness of your health condition with your relatives?**
6. Yes
7. No
8. **Do you think that you get enough information about your disease from physicians?**
9. I would like to have more information
10. I have enough information
11. I would like to have less information
12. **During care of patients with serious illness subsequent topics are often discussed. Please indicate if someone from the hospital talked with you about any of these:**
13. Hospice care YES / NO
14. Advance directives YES / NO
15. Do not resuscitate orders YES / NO

**9. What have been your main problems or concerns over the past 3 days?**

a) …………………………………………………………………………………………………………

b) …………………………………………………………………………………………………………

c) …………………………………………………………………………………………………………

**10. Below is a list of symptoms, which you may or may not have experienced. For each symptom, please tick the box that best describes how it has affected you over the past 3 days.**

|  | **Not at all** | **Slightly** | **Moderately** | **Severely** | **Overwhelmingly** |
| --- | --- | --- | --- | --- | --- |
| Pain |  |  |  |  |  |
| Shortness of breath |  |  |  |  |  |
| Weakness or lack of energy |  |  |  |  |  |
| Nausea (feeling like you are going to be sick) |  |  |  |  |  |
| Vomiting |  |  |  |  |  |
| Poor appetite |  |  |  |  |  |
| Constipation |  |  |  |  |  |
| Sore or dry mouth |  |  |  |  |  |
| Drowsiness |  |  |  |  |  |
| Poor mobility |  |  |  |  |  |

**11. Please list any other symptoms not mentioned above, and tick the box to show how they have affected you over the past 3 days.**

|  | **Not at all** | **Slightly** | **Moderately** | **Severely** | **Overwhelmingly** |
| --- | --- | --- | --- | --- | --- |
| a) |  |  |  |  |  |
| b) |  |  |  |  |  |
| c) |  |  |  |  |  |

# 12. Over the past 3 days:

|  | **Not at all** | **Occasionally** | **Sometimes** | **Most of the time** | **Always** |
| --- | --- | --- | --- | --- | --- |
| **Have you been feeling worried about his/her illness or treatment?** |  |  |  |  |  |
| **Have any of your family or friends been anxious or worried about the patient?** |  |  |  |  |  |
| **Have you been feeling depressed?** |  |  |  |  |  |

|  | **Always** | **Most of the time** | **Sometimes** | **Occasionally** | **Not at all** |
| --- | --- | --- | --- | --- | --- |
| **Have you felt at peace?** |  |  |  |  |  |
| **Have you been able to share how you are feeling with your family or friends as much as you wanted?** |  |  |  |  |  |
| **Have you had as much information you wanted?** |  |  |  |  |  |

|  | **Problems addressed/ No problems** | **Problems mostly addressed** | **Problems partly addressed** | **Problems hardly addressed** | **Problems not addressed** |
| --- | --- | --- | --- | --- | --- |
| Have any practical problems resulting from your illness been addressed? (such as financial or personal) |  |  |  |  |  |

|  | **Alone** | **With help from a friend or relative** | **With help from a member of staff** |
| --- | --- | --- | --- |
| How did you complete this questionnaire? |  |  |  |

**13.** How would you rate your overall quality of life during the past week?

| 1 | 2 | 3 | 4 | 5 | 6 | 7 |
| --- | --- | --- | --- | --- | --- | --- |

Very poor Excellent

**14. During the course of your illness it is needed to make important decisions about health care. Physicians, patient and family members are usually involved in this decision-making process. How important role should each of them play in the decision-making process?**

*Indicate the degree of importance for every participant on range 0-10 (0 means – in decision-making about health care is his/her opinion very unimportant, 10 means this opinion is very important for me.):*

| a) | **Patient** | 0 | 1 | 2 | 3 | 4 | 5 | 6 | 7 | 8 | 9 | 10 |
| --- | --- | --- | --- | --- | --- | --- | --- | --- | --- | --- | --- | --- |
| b) | **Physician** | 0 | 1 | 2 | 3 | 4 | 5 | 6 | 7 | 8 | 9 | 10 |
| c) | **Family** | 0 | 1 | 2 | 3 | 4 | 5 | 6 | 7 | 8 | 9 | 10 |

**Age:** …………………..

**Gender:**

1. male
2. female

**Education:**

1. elementary school
2. high school
3. graduate degree

**Do you consider yourself as religious?**

1. yes
2. no

**How do you evaluate answering this questionnaire?**

1. Very unpleasant
2. Unpleasant
3. I do not mind
4. Interesting
5. Very interesting
